# ACA-A1 segment development affects basal ganglia infarct severity and thrombectomy safety in MCA occlusion

**DOI:** 10.1093/esj/aakag054

**Published:** 2026-07-08

**Authors:** Wenping Sun, Guangchen He, Haitao Lu, Liming Wei, Haoyang Xu, Yueqi Zhu

**Affiliations:** Department of Radiology, Shanghai Sixth People’s Hospital Affiliated to Shanghai Jiao Tong University School of Medicine, No. 600 Yishan Road, Shanghai 200233, China; Department of Radiology, Shanghai Sixth People’s Hospital Affiliated to Shanghai Jiao Tong University School of Medicine, No. 600 Yishan Road, Shanghai 200233, China; Department of Radiology, Shanghai Sixth People’s Hospital Affiliated to Shanghai Jiao Tong University School of Medicine, No. 600 Yishan Road, Shanghai 200233, China; Department of Radiology, Shanghai Sixth People’s Hospital Affiliated to Shanghai Jiao Tong University School of Medicine, No. 600 Yishan Road, Shanghai 200233, China; Department of Radiology, Shanghai Sixth People’s Hospital Affiliated to Shanghai Jiao Tong University School of Medicine, No. 600 Yishan Road, Shanghai 200233, China; Department of Radiology, Shanghai Sixth People’s Hospital Affiliated to Shanghai Jiao Tong University School of Medicine, No. 600 Yishan Road, Shanghai 200233, China; Department of Interventional Radiology, Fengxian Hospital, The Third School of Clinical Medicine, Southern Medical University, Shanghai, China

**Keywords:** anterior cerebral artery, basal ganglia infarction, lenticulostriate artery, thrombectomy

## Abstract

**Introduction:**

The lenticulostriate arteries (LSAs) demonstrate developmental variability that is linked to the A1 segment of the anterior cerebral artery (ACA) anatomy. In acute ischaemic stroke (AIS) with MCA occlusion, basal ganglia (BG) infarction extent and haemorrhagic transformation (HT) correlate with LSA involvement. This study aimed to assess whether ipsilateral A1-ACA variations influence BG infarction severity and thrombectomy outcomes.

**Patients and methods:**

A control cohort (*n* = 300) undergoing catheter angiography from September 2023 to November 2024 was used to define anatomical relationships between the A1-ACA and the medial LSA (mLSA). A thrombectomy cohort (*n* = 236; MCA occlusion) from January 2019 to November 2024 was stratified by A1-ACA status (normal vs hypoplastic/absent) on pre-intervention CTA. Final basal ganglia infarct volume (FIV_BG_) was quantified on 72-h post-procedure MRI/NCCT. Associations between A1-ACA status, FIV_BG_, HT and outcomes were analysed via multivariable regression. Mediation analysis evaluated FIV_BG_’s role in A1-ACA anatomy and HT relationships.

**Results:**

Hypoplastic/absent A1 segments occurred in 16.3% (49/300) of controls, who had a reduced mLSA presence (6.1% vs 20.7%, *P =* .016) and increased predominant lateral LSAs (71.4% vs 50.6%, *P =* .007) compared to those with a normal A1. Among thrombectomy patients, hypoplastic/absent A1 (15.3%, 36/236) predicted larger FIV_BG_ (median: 29.01 cm^3^ [24.4–37.8] vs median: 25.80 cm^3^ [21.5–32.5], *P =* .004) and higher HT rates (66.67% vs 34%, *P* < .01). Multivariable analyses confirmed A1 hypoplasia/absence was independently associated with FIV_BG_ (β = 5.714; 95% CI, 2.860–8.569; *P* < .01) and HT (aOR = 3.059; 95% CI, 1.284–7.288; *P* = .012). Furthermore, FIV_BG_ mediated 44% of the indirect effect of A1 development on HT post-EVT.

**Conclusions:**

Hypoplastic/absent A1 segments correlate with impaired mLSA development, thereby exacerbating BG infarction and HT risk post-thrombectomy. As one of several predictors, A1 segment morphology on preprocedural CTA may help stratify patients at risk for larger BG infarcts and haemorrhagic complications.

## Graphical Abstract

**Graphical Abstract:**
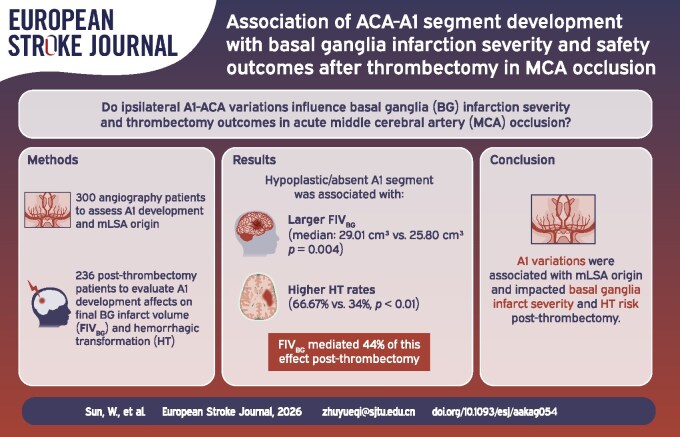

## Introduction

Acute MCA occlusion is a leading cause of ischaemic stroke, accounting for significant morbidity and mortality.^1^ Endovascular thrombectomy (EVT) is the standard treatment for LVO in the anterior circulation, with recanalisation rates of 80%–90%. However, fewer than 50% of patients achieve favourable functional outcomes.^2^^,^^3^ This discrepancy underscores the prognostic importance of infarct topology alongside infarct volume.^4^ The basal ganglia (BG), a region highly vulnerable to ischaemic injury because of its unique metabolic and vascular anatomy,^5^ are particularly prone to haemorrhagic transformation (HT) following infarction,^6–8^ which exacerbates poor clinical outcomes.^6^

The lenticulostriate arteries (LSAs), delicate perforators supplying the BG, are critically involved in cerebrovascular pathologies such as lacunar infarctions and deep haemorrhages.^9^ Anatomically, the LSAs are classified into medial (mLSA) and lateral groups, with mLSAs exhibiting marked interindividual variability.^10–13^ Medial LSAs predominantly arise from the A1 segment of the anterior cerebral artery (A1-ACA) or the M1-MCA, and perfuse key BG subregions, including the caudate head, anterior internal capsule and anteroinferior putamen/pallidum.^10^^,^^12^^,^^13^ Embryologically, shared developmental origins between mLSAs and the A1-ACA underlie their anatomical codependence.^14^ Notably, A1-ACA hypoplasia or absence, observed in 17.8% of ischaemic stroke patients,^15^ may render BG perfusion predominantly MCA-dependent, increasing infarction volume during MCA occlusion.^13^^,^^16^

Despite these insights, the clinical implications of A1-ACA variants on mLSA development and their downstream effects on BG infarction and HT remain poorly characterised. We hypothesise that A1-ACA anatomical variations dictate mLSA morphology, thereby affecting BG ischaemic injury post-EVT. Elucidating these relationships could yield imaging biomarkers to predict EVT outcomes and optimise therapeutic strategies.

## Patients and methods

### Study design and patients

This study was approved by the institutional review board. Two patient cohorts were assessed at our centre. First, a control cohort was used to evaluate the relationship between anatomical variations in the A1-ACA and the mLSAs. This cohort included patients who underwent catheter angiography between September 2023 and November 2024. The exclusion criteria were occlusion or more than 50% stenosis of the A1-ACA or MCA and suspected lenticulostriate infarction.

Second, a thrombectomy cohort included consecutive patients with acute ischaemic stroke (AIS) and MCA occlusion who underwent EVT at our institution from January 2019 to November 2024. Inclusion criteria were: (1) available pre-interventional imaging including non-contrast CT (NCCT) and CTA; (2) isolated proximal M1 MCA occlusion; (3) successful recanalisation with a modified treatment in cerebral ischaemia (mTICI) score ≥ 2b and (4) NCCT within 24 h or MRI within 3 days post-treatment. Patients with inadequate imaging quality were excluded.

### CT acquisition protocol

Non-contrast CT and CTA were conducted using either a 64-section CT scanner (Brilliance 64, Philips Healthcare) set at 120 kV, 333 mA, and a 0.75-s rotation, or a 640-section CT scanner (United Imaging Healthcare, Shanghai, China) set at 120 kVp, 300 mAs, and a 0.5-s rotation. An intravenous contrast agent, iopromide (60–100 mL, 370 mg iodine/mL; Ultravist 370, Bayer), was administered at a rate of 4 mL/s. Scanning was initiated when CT attenuation in the ascending aorta reached 120 HU. Volume rendering and full-slab maximum intensity projection (MIP) images were processed using a multimodality workstation (Philips Medical Systems, Philips Healthcare) with 0.67-mm axial sections, reconstructed at 0.8-mm increments.

### Cerebral angiography procedure

Cerebral angiography was performed with a digital angiography unit (Artis zee or Q, Siemens Healthineers, Erlangen, Germany) under local anaesthesia and intravenous conscious sedation. A diagnostic catheter was placed in the proximal internal carotid artery (ICA) for two-dimensional (2D) digital subtraction angiography (2D-DSA) in anterior–posterior and lateral projections. For further evaluation, 3D DSA was utilised. Rotational scans followed a standard protocol: separate native mask and contrast-enhanced runs, each lasting 5 s. These scans produced 133 projected images with a 200° rotation angle and a detector dose of 0.36 μGy/image (70 kV, 1024 × 1024 detector array, 16 cm × 16 cm projection size, 1.5°/frame increment, 30 frames/s). All 3D image data were processed on a syngo X-Workplace (VD20B; Siemens Healthcare GmbH, Forchheim, Germany), utilising prototype software dedicated to clinical and research applications. The 3D DSA reconstructions from fill and native runs helped diagnose anatomical variations in A1-ACA development and mLSA origination in the control cohort.

### Thrombectomy procedure

The stent retriever and catheter aspiration combination technique was used as the primary approach for thrombectomy. After a 90-cm sheath (Terumo, Tokyo, Japan) was introduced into the cervical segment of the ICA on the affected side, an aspiration catheter (Penumbra, Alameda, CA, USA) was subsequently advanced into the distal ICA with a microcatheter over a microwire. The microcatheter was positioned distal to the thrombus, allowing the deployment of a stent retriever (Solitaire, ev3 Neurovascular, Covidien, Irvine, CA, USA) in accordance with the diameter of the occluded artery (typically 6 × 30 mm or 4 × 20 mm for proximal M1 segment occlusions). An integration period of 3–5 min was allocated for clot integration. While using the stent retriever as an anchor, the aspiration catheter was manoeuvred up to a position just proximal to the stent to enhance the aspiration efficacy. The stent retriever was then withdrawn into the aspiration catheter and removed from the entire system. The aspiration catheter was subsequently removed under continuous aspiration and proximal flow arrest to capture potential micro-fragments of the thrombus. This retrieval attempt was repeated up to 3 times per target artery. In cases of technical failure, single-catheter aspiration or stent thrombectomy was utilised. Antegrade reperfusion was assessed using the mTICI grade. Successful reperfusion was defined as an mTICI grade of 2b-3 after EVT.

### Imaging analysis

**Figure 1:**
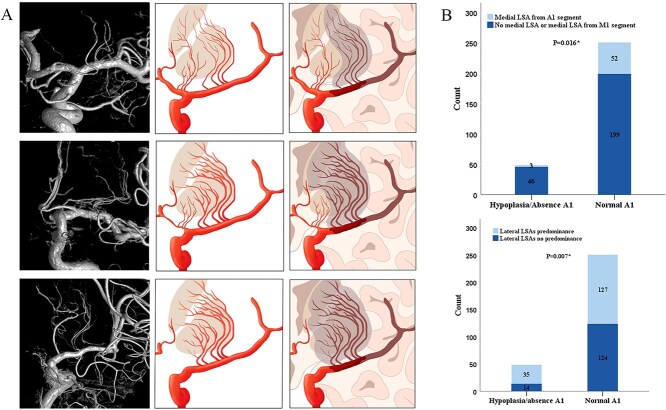
Classification and distribution of the LSAs among groups with hypoplastic/absent A1 segments and those with normal A1 segments. (A) Three-dimensional DSA datasets alongside an illustration detailing the LSA classification and the associated pattern of lenticulostriate infarction. (B) Individuals with hypoplastic or absent A1 segments have a significantly reduced presence of mLSAs (*P =* .016) and a higher prevalence of predominant lateral LSAs (*P =* .007) compared to those with normal A1 segments. Comparisons were performed using Pearson’s chi-square test. Abbreviations: DSA = digital subtraction angiography; LSAs = lenticulostriate arteries; mLSAs = medial lenticulostriate arteries.

The cerebral vasculature was assessed using 2D and 3D DSA in the control cohort. In the thrombectomy cohort, A1 segments were first assessed on thin-slice multiplanar CTA, and final classification was confirmed on intraoperative or postoperative DSA to minimise bias from transient haemodynamic impairment. Anatomical variations in the A1 segment were categorised as normal, absent or hypoplastic. A hypoplastic A1 segment of the ACA was defined as a diameter reduction of more than 50% compared to the contralateral side.^17^ In the control cohort, the arteries that supplied blood to the BG in the anterior circulation were classified based on the origin sites of the dominant branches, following the Yaşargil classification.^11^^,^^18^ The arteries were divided into 3 categories as shown in Figure 1A: (1) the recurrent artery of Heubner (RAH), primarily originating from the A1-A2 junction; (2) the mLSA, typically originating from the A1-ACA and (3) the lateral LSA, originating from the M1 segment of the MCA (M1-MCA). If the mLSA was underdeveloped or absent or originated from the M1 segment, lateral LSAs were considered the predominant source of BG blood supply (Figure 1A). Two experienced neuroradiologists, each with more than 10 years of expertise, evaluated the LSA and the A1 segment. Both were blinded to the patients’ clinical characteristics, and decisions were made by consensus. The degree of inter-rater agreement was assessed.

The collateral circulation was assessed using baseline CTA source images and MIP reconstructions, with a 0–3 grading scale adopted from Tan et al.^19^ The Alberta Stroke Program Early CT Scores (ASPECTS) were calculated from post-EVT NCCT or MRI scans. The Alberta Stroke Program Early CT Scores for the basal ganglia (ASPECT_BG_)—encompassing the caudate, lentiform nucleus and internal capsule—were employed for a semiquantitative assessment of BG infarctions. The final ischaemic volume of the basal ganglia (FIV_BG_) was evaluated using DWI sequences (*b* value of 1000) from MRI. In instances where MRI was unavailable, follow-up NCCT served as an alternative for assessing the FIV_BG_. Data were transferred to a dedicated workstation (Syngo.via, VB 40; Siemens Healthineers, Germany). Two neuroradiologists, blinded to clinical and angiographic data, independently delineated the FIV in the BG slice by slice and performed semiautomatic measurements on the workstation. The final FIV_BG_ value was determined by averaging the 2 measurements.

### Statistical analysis

Statistical analyses were performed using SPSS version 24.0 (IBM, Armonk, NY) and R software (version 4.0.3; R Foundation for Statistical Computing). A *P*-value < .05 was considered to indicate statistical significance. Normal distribution was assessed using the Kolmogorov–Smirnov test. Continuous variables are presented as mean ± SD or median with IQR, as appropriate, while categorical variables are expressed as percentages. Group comparisons for continuous variables were conducted using the Mann–Whitney *U* test, and the χ^2^ test for categorical variables. Multivariate logistic regression analyses were performed to identify independent factors associated with HT. The events-per-variable (EPV) ratio was calculated to evaluate model stability and the risk of overfitting. Given the small sample size of the hypoplastic/absent A1 group, Firth’s penalised logistic regression with 1000 bootstrap resamples^20^ was also performed to mitigate small-sample bias. Variables with *P*-values < .1 in the univariate analysis were included in the final multivariate logistic regression model. Interrater agreement was measured using Cohen’s kappa coefficient and the intraclass correlation coefficient (ICC).

To identify independent predictors for the FIV_BG_, baseline demographic data and stroke characteristics were subjected to univariate analyses. For categorical variables, the Mann–Whitney *U* test and Kruskal–Wallis tests were applied, while Spearman’s correlation coefficient was used for continuous variables. Variables with *P* values < .05 in the univariate analysis as well as those believed to have an important influence on the dependent variable were included in a multiple linear regression model to assess their association with FIV_BG_. Receiver operating characteristic curve analyses were performed to identify the discriminatory performance of FIV_BG_ for predicting HT risk. Internal validation using 1000 bootstrap resamples^21^ was performed to calculate the bias-corrected area under the curve (AUC) values.

The mediation analysis was performed using the product-of-coefficients method^22^ to assess whether FIV_BG_ mediates the association between A1 development and HT risk. Through this approach, the effect of A1 development on FIV_BG_ (Za) and the effect of FIV_BG_ on HT risk while adjusting for A1 development (Zb) were estimated and the significance of their product (Za × Zb) was evaluated. A 95% CI excluding 0 was considered to indicate statistical significance. Rho-parameterised sensitivity analysis^23^ was conducted to evaluate unmeasured confounding robustness.

## Results

### ACA-A1 variation and medial LSA development in the control cohort

**Figure 2:**
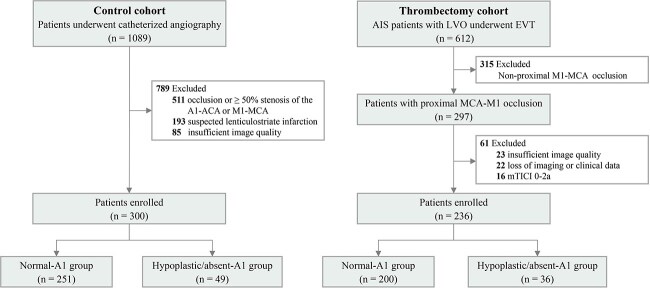
Flow diagram of the current dual-cohort study. Abbreviations: AIS = acute ischaemic stroke; ACA = anterior cerebral artery; LSAs = lenticulostriate arteries.

The control cohort for investigating mLSA development comprised 300 patients, including 177 males and 123 females, with a mean age of 59.5 years (range: 22–92 years) (Figure 2). Angiographic analysis revealed 251 (83.7%) patients with normal A1-ACAs, whereas 49 (16.3%) displayed hypoplastic or absent A1-ACAs. Detailed anatomical information about mLSAs was obtained through 2D and 3D DSA, resulting in high interrater agreement (Cohen’s κ = 0.87; 95% CI, 0.788–0.960). The presence of mLSAs was significantly lower in the hypoplastic/absent A1 group than in the normal A1 group (6.1% [3/49] vs 20.7% [52/251]; *P =* .016). Conversely, lateral LSA predominance was significantly greater in the hypoplastic/absent group (71.4% [35/49] vs 50.6% [127/251]; *P* = .007). The frequency of large RAHs was similar between the groups (22.5% [11/49] vs 28.7% [72/251]; *P* = .372) (Figure 1B).

### Patients in the thrombectomy cohort

**Figure 3:**
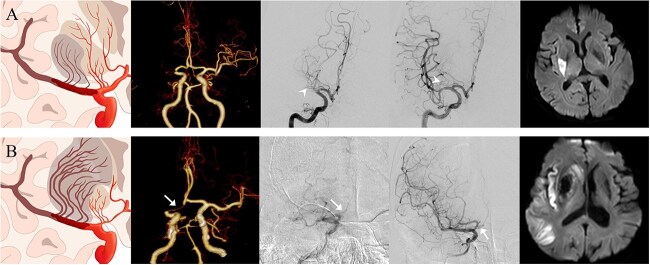
Examples of the normal and hypoplastic A1 segments in patients who developed basal ganglia infarction within the proximal M1 segment of the MCA occlusions treated via thrombectomy. (A) Initial CTA and DSA showing the normal A1 segment. Initial DSA image showing prominent medial LSAs from the proximal A1 segment (white arrowheads), which give rise to small arteries supplying the basal ganglia. DWI within 72 h after EVT (mTICI 3, with SOR-T 300 min) revealed restricted infarction in the lentiform nucleus. (B) Initial CTA and DSA showing the hypoplastic A1 segment (white arrows). Diffusion-weighted MR imaging (DWI) within 72 h after EVT (mTICI 3, SOR-T 320 min) revealed restricted infarction in the caudate, lentiform nucleus and cortex, with haemorrhagic transformation. Abbreviations: DSA = digital subtraction angiography; mTICI = modified treatment in cerebral ischaemia; SOR-T = symptom onset to recanalisation time.

A total of 612 patients with MCA occlusion who underwent EVT were assessed, with 236 ultimately included in the thrombectomy cohort (Figure 2). The 2 researchers demonstrated good interobserver agreement for the A1-ACA classification (Cohen’s κ = 0.89; 95% CI, 0.840–0.942) and collateral score (ICC = 0.84; 95% CI, 0.796–0.878). Agreement for A1 segment classification between baseline CTA and combined intraoperative DSA was substantial for the 2 researchers (Cohen’s κ = 0.82; 95% CI, 0.723–0.918 and κ = 0.86; 95% CI, 0.773–0.938, respectively). In thrombectomy cohort, 15.3% (36/236) had a hypoplastic (*n* = 31) or absent (*n* = 5) A1 segments (hypoplastic/absent-A1 group). Compared with the normal-A1 group, this group had a higher incidence of hypertension (75% [27/36] vs 57.5% [115/200]; *P =* .048) and exhibited a greater FIV_BG_ compared to those with normal A1 development (median: 29.01 cm^3^ [24.4–37.8] vs median: 25.80 cm^3^ [21.5–32.5]; *P =* .004; Table 1 and Figure 3). The other baseline factors did not significantly differ between the groups, as detailed in Table 1. Follow-up imaging modalities including DWI in 88.6% patients (209/236) and NCCT in 11.4% (27/236). Among the 16 patients excluded due to unsuccessful recanalisation, there was no significant difference in the prevalence of A1 segment hypoplasia or absence when compared to the thrombectomy cohort that achieved successful recanalisation (6.3% [1/16] vs 15.3% [36/236]; *P =* .325).

**Table 1:** Baseline characteristics based on different A1 anatomical variations.

Characteristic	Overall	A1 anatomical variations		*P* value
		Normal	Hypoplastic/Absent	
	*n* = 236	*n* = 200	*n* = 36	
**Sex, female**	101 (42.80)	88 (44)	13 (36.11)	.378
**Age (years)**	72 (62, 82)	72 (62.3, 82)	70 (59.8, 82)	.407
**Risk factors**				
**Hypertension**	142 (60.17)	115 (57.50)	27 (75.00)	.048
**Diabetes**	104 (44.07)	87 (43.50)	17 (47.22)	.679
**Atrial fibrillation**	90 (38.14)	80 (40.00)	10 (27.78)	.165
**Coronary artery disease**	54 (22.88)	48 (24)	6 (16.67)	.335
**Baseline NIHSS**	15 (12, 18)	15 (12, 18)	16 (10.8, 19.5)	.812
**Collateral score**	2 (1, 2)	2 (1, 2)	2 (1, 2)	.515
**IVT**	91 (38.56)	78 (39.00)	13 (36.11)	.743
**Symptom-onset to final recanalisation (min)**	290 (270, 315)	290 (275, 315)	280.5 (265, 320)	.229
**Post-EVT ASPECTS**_**BG**_	2 (1, 2)	2 (1, 2)	1 (0.3, 2)	.012
**FIV**_**BG**_ **(cm**^**3**^**)**	26.34 (21.73, 33.8)	25.80 (21.5, 32.5)	29.01 (24.4, 37.8)	.004
**Haemorrhagic transformation**	92 (38.98)	68 (34.00)	24 (66.67)	<.01
**mRS score ≤ 2 at 90 days**	157 (66.53)	137 (68.50)	20 (55.56)	.13

Data are expressed as *n* (%); median (Q1, Q3). ASPECTS_BG_ = Alberta Stroke Program Early CT Score of BG regions; IVT = intravenous thrombolysis; FIV_BG_ = final infarct volume in basal ganglia; HT = haemorrhagic transformation.

### Correlation of risk factors with FIV_BG_

Beyond the notable correlation between A1 segment development and the FIV_BG_ (as presented in Table 1 and Table S1), Spearman’s correlation analysis revealed a significant association between baseline NIHSS scores and FIV_BG_ (*r_s_* = 0.524; *P* < .01; Table S2). Furthermore, multiple linear regression analysis revealed key predictors for a larger FIV_BG_: a hypoplastic/absent A1 segment (β = 5.714; 95% CI, 2.860–8.569; *P* < .01) and a higher baseline NIHSS score (β = 0.833; 95% CI, 0.650–1.017; *P* < .01; Table 2). Symptom-onset-to-recanalisation time showed a borderline association with FIV_BG_ (β = 0.030; 95% CI, −0.000 to 0.061; *P =* .051; Table 2).

**Table 2:** Multiple linear regression to assess correlations between risk factors and FIV_BG_.

Variable	*B*	*t* value	*P* value	95% CI
**Hypoplastic/absent A1**	5.714	3.945	<.01	2.860–8.569
**Baseline NIHSS**	0.833	8.928	<.01	0.650–1.017
**Symptom-onset to final recanalisation (min)**	0.030	1.965	.051	−0.000–0.061

Abbreviation: FIV_BG_ = final infarct volume in basal ganglia.

Dependent variable: FIV_BG_; *R*^2^ = 0.300, adjusted *R*^2^ = 0.291, *F* = 33.166, *P* = .000.

### Association of A1 development with clinical outcome

There was no significant difference in 90-day functional outcomes, assessed by the mRS ≤ 2, between patients with normal A1 segments (68.5% [137/200]) and those with hypoplastic or absent A1 segments (55.56% [20/36]; *P* = .13; Table 1). However, HT rates were significantly higher in the hypoplastic/absent-A1 group (66.67% [24/36] vs 34% [72/200]; *P* < .01; Table 1 and Table S3), with absent A1 present in 3 affected patients. In terms of specific HT types, compared with the normal A1 group, the hypoplastic/absent-A1 group had a significantly higher rates of haemorrhagic infarction (44.5% [16/36] vs 24% [48/200]; *P* < .05) and parenchymal haematoma (22.2% [8/36] vs 10% [20/200]; *P* < .05) compared to the normal A1 group (Figure S1).

The EPV ratio of 18.4, exceeding the recommended threshold of 10. Multivariable analysis adjusted for confounders revealed that A1 hypoplasia or absence (adjusted OR: 3.059; 95% CI, 1.284–7.288; *P* = .012) and FIV_BG_ (adjusted OR: 1.2; 95% CI, 1.130–1.274; *P* < .01) were independently associated with HT risk (Table 3). Firth’s penalised logistic regression with 1000 bootstrap resamples confirmed that A1 hypoplasia or absence remained an independent risk factor for HT (adjusted OR: 2.944; 95% CI, 1.244–6.963; *P* = .014; Table S4).

**Table 3:** Multivariate logistic regression to predict HT following EVT.

Variable	*P* value	OR (95% CI)
**Hypoplastic/absent A1**	.012	3.059 (1.284–7.288)
**FIV**_**BG**_	<.01	1.2 (1.130–1.274)
**Post-EVT ASPECTS**_**BG**_	.039	1.783 (1.030–3.088)
**Baseline NIHSS**	.637	0.984 (0.919–1.053)
**IVT**	.205	0.650 (0.333–1.266)

Abbreviations: ASPECTS_BG_ = Alberta Stroke Program Early CT Score of BG regions; FIV_BG_ = final infarct volume in basal ganglia; HT = haemorrhagic transformation; IVT = intravenous thrombolysis.

Final ischaemic volume of the basal ganglia demonstrated significant discriminative ability for HT (AUC = 0.785, 95% CI, 0.725–0.845; *P* < .001). At the optimal cutoff value of 32.86 cm^3^, the model achieved a specificity of 90.3% and a sensitivity of 54.3%. Bootstrap validation confirmed robust and stable predictive performance (corrected AUC = 0.782, 95% CI, 0.721–0.845; Figure S2).

In the sensitivity analyses, excluding A1 absence, A1 hypoplasia remained independently associated with FIV_BG_ (β = 6.425; 95% CI, 3.394–9.457; *P* < .01; Table S5) and with HT (adjusted OR 2.959; 95% CI, 1.176–7.442; *P* = .021; Table S6). After excluding those with follow-up NCCT (*n* = 27), FIV_BG_ remained independently associated with A1 hypoplasia (β = 6.791; 95% CI, 3.758–9.824; *P* < .01; Table S7) and with HT (adjusted OR: 1.258; 95% CI, 1.165–1.357; *P* < .01; Table S8).

### Mediation of A1 development effect on HT by FIV_BG_

Mediation analysis was conducted with HT as the dependent variable, A1 development as the independent variable, and FIV_BG_ as the mediator. The analysis revealed a significant indirect effect of A1 development on HT risk through FIV_BG_ (*B* = 0.868; SE = 0.297; 95% CI, 0.340–1.507; Table S9). This mediation accounted for 44% of the total effect. The sensitivity analysis demonstrated significant robustness to unmeasured confounding, with an average causal mediation effect of 1.146 (95% CI, 0.494–2.165; *P* = .001) that would be nullified by a confounding effect exceeding ρ = 0.006 (Figure S3).

## Discussion

In this study, the impact of A1 segment development on the occurrence of mLSAs and their role in stroke outcomes was investigated. Using a control cohort, we confirmed that patients with hypoplastic or absent A1 segments exhibit a significantly lower presence of mLSAs compared to those with normal A1 development. In the thrombectomy cohort, hypoplastic or absent A1 segments were associated with an increased susceptibility to BG infarction during MCA occlusion. Despite similar clinical outcomes at 90 days post-successful recanalisation, patients with these A1 variations had higher rates of HT than those with normal A1 segments. Further mediation analysis demonstrated that FIV_BG_ significantly mediated the indirect effect of A1 development on HT following EVT.

The anatomical variability and clinical relevance of LSAs have been extensively studied through regional anatomy and advanced in vivo imaging techniques.^10–13^^,^^24–28^ Previous research has revealed a shared embryogenetic origin between mLSAs and the ACA, suggesting interrelated anatomical variations.^14^ However, the precise relationship between the A1 segment and mLSAs remains unclear. Digital subtraction angiography, the gold standard for imaging LSAs, has been limitedly applied in this context.^11^^,^^12^ In our current study, we utilised dynamic 2D and 3D DSA imaging to assess the origins and development of LSAs in association with A1 segment variations. We observed a low prevalence (18.3%) of mLSAs and a high prevalence (54%) of lateral LSA dominance, consistent with previous findings.^12^^,^^13^ Further analysis revealed that the presence of hypoplasia or absence of the A1 segment significantly reduced the occurrence of mLSAs and notably increased the dominance of lateral LSAs. Medial and lateral LSAs appear to maintain a balance in supplying the BG. When medial LSAs are underdeveloped, lateral LSAs compensate and vice versa.^14^ Occasionally, a large RAH can substitute for the mLSA’s role in BG blood supply,^13^ regardless of variations in A1 development. This highlights a correlation between A1 hypoplasia or absence and diminished BG blood supply by mLSA. Consequently, in cases of MCA obstruction with concurrent A1 hypoplasia or absence, the impact on regions primarily supplied by lateral LSAs is pronounced.

To verify the above hypothesis, another AIS-LVO cohort was enrolled to assess the impact of A1 segment development on ischaemic outcomes in the BG. Several studies have investigated the influence of anatomical variations in the A1 segment on AIS.^17^^,^^29–32^ However, research on the relationship between A1 variants and BG infarction remains scarce. Chuang et al.^16^ described the phenomenon whereby A1 hypoplasia appears to be a risk factor for ischaemic stroke in the BG, as the A1 segment gives rise to perforating arteries that supply this region; however, a systematic analysis was not performed. Our study revealed that the FIV_BG_ was significantly greater in patients with AIS with a hypoplastic A1 segment than in those with a normal A1 segment. These findings quantitatively demonstrated a significant relationship between A1 development and lenticulostriate infarctions and confirmed the role of the mLSA originating from A1 segment in supplying blood to the BG. In contrast to our findings, Feekes et al.^33^ reported a limited contribution of the perforators derived from the ACA to the BG by evaluating a group of specimens. This discrepancy may be due to the small sample size (40 hemispheres) and the lack of a quantitative analysis in this regional anatomy study. Previous studies have indicated that the origin of the LSAs and the exact location of the occlusion site are crucial to the ischaemic fate of the BG.^18^^,^^34^^,^^35^ However, LSAs exhibit large individual variability and evaluating them directly in the emergency setting remains a great challenge. Consequently, the development of the A1 might serve as a dependable biomarker for predicting the ischaemic outcomes of BG in clinical settings.

The evaluation of BG infarction currently lacks standardised criteria. The ASPECTS, a semi-quantitative method for assessing infarcted areas in the anterior circulation, is used to understand ischaemic topology.^4^^,^^36^ The BG regions, including the caudate, lentiform nucleus and internal capsule, receive blood supply from LSAs and lack collateral circulation.^33^^,^^37^ Infarcted BG demonstrates specific topologies based on the involvement of distinct LSAs,^34^^,^^38^ thus, ASPECTS can theoretically be applied to quantify BG infarction (ASPECTS_BG_). In this study, patients with A1 hypoplasia or absence typically had lower post-EVT ASPECTS_BG_ scores, indicating more extensive infarction and severe LSA branch involvement. These findings confirm that BG blood supply relies mainly on LSAs originating from the MCA in such patients, which is consistent with the anatomical variations observed in the control cohort. Final ischaemic volume of the basal ganglia is an objective metric for quantifying the severity of BG infarction. Basal ganglia infarction exhibits time dependency owing to limited collateral circulation.^39^ Prior study suggested an approximately 100-min threshold for irreversible injury to the caudate and lentiform nuclei.^18^ In our multivariate analysis under standardised recanalisation conditions (median > 100 min), A1 hypoplastic/absent (β = 5.714; *P* < .001) was a stronger determinant of FIV_BG_ than onset-to-recanalisation time (β = 0.030; *P* = .051). These findings suggest that, in contemporary practice, baseline vascular anatomy may outweigh temporal factors in shaping BG infarction outcomes. Given the higher prevalence of reliance on LSAs originating from the MCA in patients with A1 segment hypoplasia or absence, a proximal MCA obstruction typically affecting all LSAs could significantly impact the BG. Consequently, detecting A1 hypoplasia or absence on baseline CTA in the acute proximal MCA occlusion cohort necessitates expedited treatment decisions.

In our study, we observed no statistically significant differences in the mRS scores at 3 months among different A1 segment development groups. However, we identified an association between A1 development, FIV_BG_ and HT risk. Mediation analysis suggests that A1 development directly affects HT and indirectly affects it via the mediating variable, FIV_BG_. Previous studies have shown a link between pre-treatment subcortical infarct volume and HT incidence following successful EVT.^7^ Our findings support this relationship and highlight the importance of subcortical infarct volume in evaluating the risk of HT after treatment. In addition, our research explored anatomical markers related to infarct volume, notably the association between the A1 segment development and mLSAs. We hypothesise that a greater number of involved LSAs is associated with larger infarct volume and more severe reperfusion injury in the BG region. However, the pathogenesis of HT in BG infarction involves complex, multifactorial mechanisms that warrant further systematic investigation. These findings have substantial clinical implications. Understanding the origin of LSAs is crucial, particularly before EVT procedures. In ischaemic stroke patients with MCA occlusion, detecting hypoplastic or absent A1 segments on initial CTA necessitates expedited endovascular intervention, careful postoperative blood pressure management, rigorous antithrombotic therapy, and vigilant imaging follow-up. This information enables more cautious procedures to protect LSAs during interventions, especially those involving stent placement.

This study has several limitations. First, as a single-centre retrospective study, the data may contain biases or shortcomings that could impact the accuracy and reliability of our findings. Second, the data structure was asymmetrical, especially the data on patients with absent and hypoplastic A1 segments, with a relatively small sample size. Mediation analysis was limited by the small, and unbalanced sample. Unmeasured confounding may have influenced both FIV_BG_ and HT, potentially biasing the estimated mediator–outcome association. Therefore, these findings should be interpreted with caution. Given the absence of a consensus definition for A1 hypoplasia, our binary cutoff (>50% diameter reduction) is anatomically arbitrary and lacks direct validation against functional perfusion deficit. Additionally, due to the rarity of absent A1, we did not analyse it separately, instead combining it with hypoplastic A1, which may involve variations in the mLSAs. Larger-scale research is required going forward. Third, the modest explained variance in FIV_BG_ underscores the need for future studies that incorporate additional predictors such as advanced perfusion parameters, thrombus characteristics and burden of cerebral small vessel disease. Fourth, FIV_BG_ was measured on DWI or CT within 72 h in accordance with clinical practice rather than an optimised research protocol, which may overestimate infarct volume because of oedema. Moreover, the inclusion of patients with HT in the final infarct volume measurements could potentially exaggerate the FIV values. Although we sought to mitigate this by using MRI for infarct volume calculation or follow-up CT imaging beyond 72 h for patients lacking MRI, this limitation remains unavoidable. Finally, in the thrombectomy cohort, 2D image overlap limited the accurate assessment of the mLSAs.

## Conclusion

Our study revealed that variants of the A1 segment are closely associated with the development of mLSA, significantly impacting the severity of BG infarction. These findings suggest that A1 development may serve as a potential novel imaging predictor for assessing ischaemic outcomes in the BG during MCA occlusion. As tissue viability is multifactorial, further large-scale and long-term prospective studies incorporating multiparametric predictive models are warranted to validate these results.

## Supplementary Material

Supplementary_Figure_and_Table_aakag054

## Data Availability

Data are available upon reasonable request to the corresponding authors.
