# Supplementary material for: ACA-A1 segment development affects basal ganglia infarct severity and thrombectomy safety in MCA occlusion
Source: Eur Stroke J. 2026 Jul 8;11(7):aakag054. doi: 10.1093/esj/aakag054 (PMC13344103; doi:10.1093/esj/aakag054)
Supplement: Supplementary_Figure_and_Table_aakag054 [file supplementary_figure_and_table_aakag054.docx]

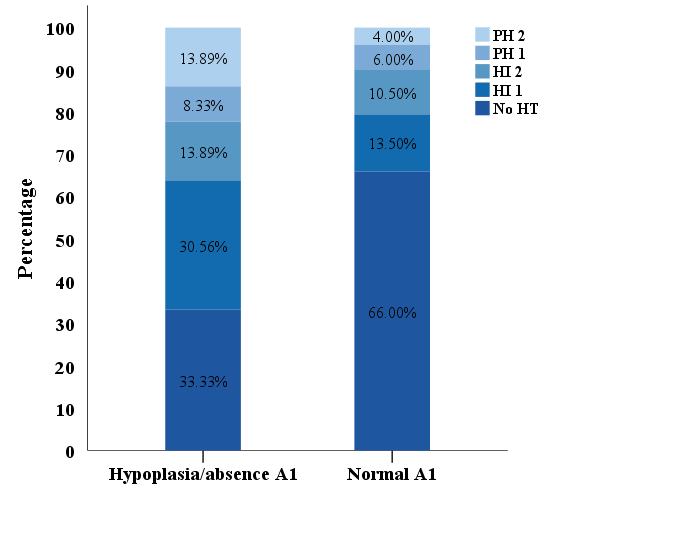


**Supplementary Figure 1: Distribution of hemorrhagic transformation subtypes in patients with hypoplasic or absent A1 segment and normal A1 segment.** Hemorrhagic transformation subtypes were classified according to the European Cooperative Acute Stroke Study criteria (ECASS). HT: hemorrhagic transformation; HI: hemorrhagic infarction; PH: parenchymal hematoma.


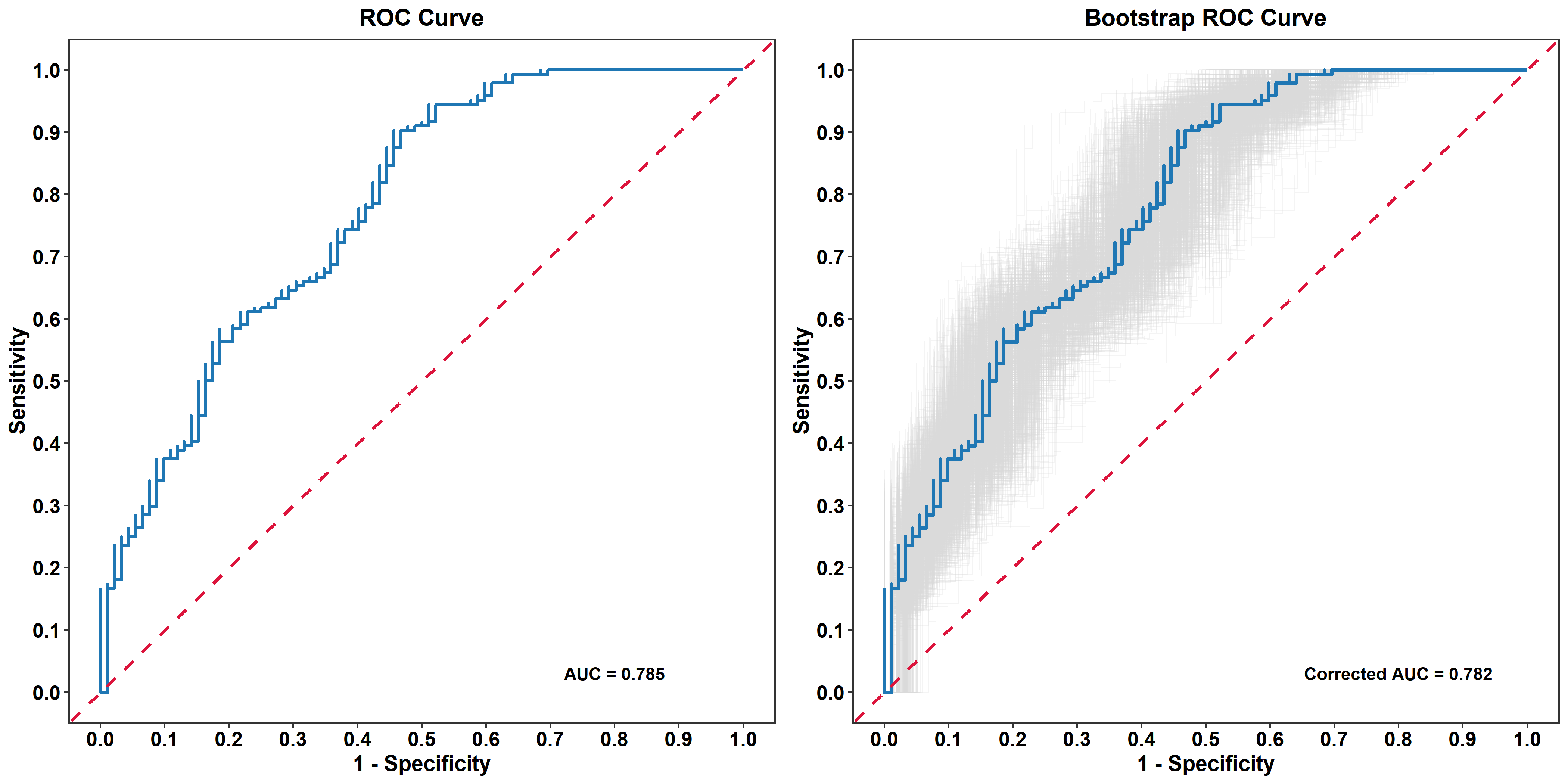


**Supplementary Figure 2:** **Receiver operating characteristic (ROC) curves of FIV_BG_ for predicting hemorrhagic transformation.** Observed ROC curve in the original cohort (AUC = 0.785, 95% CI 0.725–0.845); bootstrap ROC curve derived from 1000 bootstrap resamples (**corrected AUC = 0.782, 95% CI 0.721–0.845**).


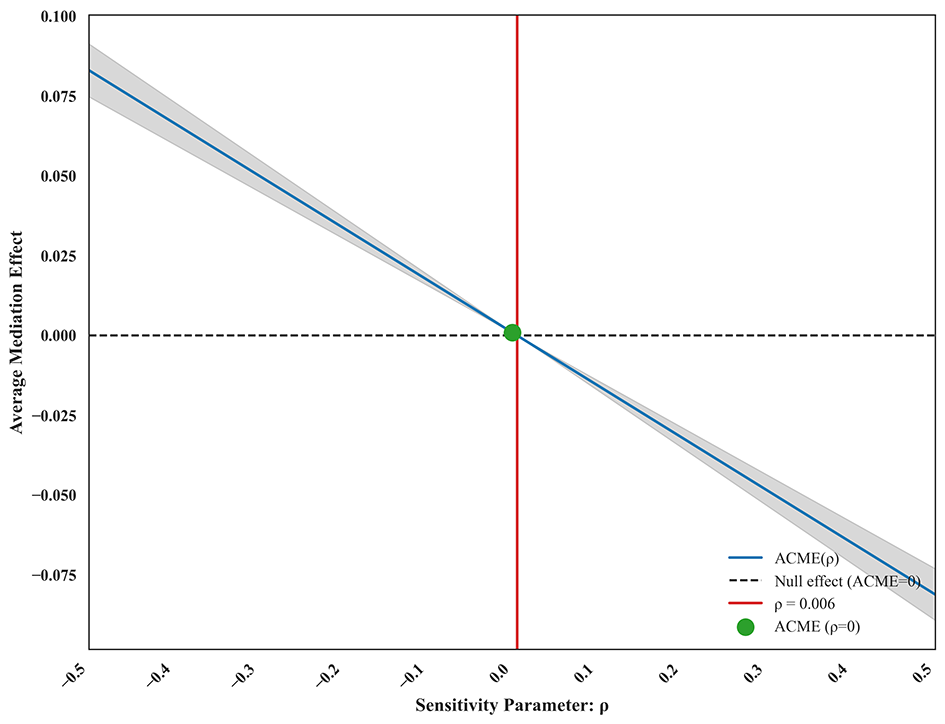


**Supplementary Figure 3: Sensitivity analysis of the indirect effect using the parameterized rho (ρ) method.** The blue line shows the average causal mediation effect (ACME) as a function of ρ (the residual correlation between the mediator and the outcome). The black dashed line denotes the null effect (ACME = 0), and the vertical red line indicates the critical ρ threshold (ρ = 0.006) where the ACME becomes null. The green dot represents the ACME at ρ = 0 (assuming no unobserved confounding), with the grey shaded area showing the 95% confidence interval (95% CI 0.494-2.165).

| **Supplementary Table 1. Association of baseline characteristics with FIV_BG_** | | | |
| --- | --- | --- | --- |
| **Variable** | **FIV_BG_** | **Z** | ***p* value** |
| Total | 26.34 (21.8, 33.8) |  |  |
| **Sex** |  | -0.188 | 0.851 |
| Female | 26.64 (21.4, 34) |  |  |
| Male | 26.27 (22, 32.9) |  |  |
| **Hypertension** |  | -0.294 | 0.769 |
| no | 27.14 (21.1, 33.5) |  |  |
| yes | 26.30 (22.2, 34.6) |  |  |
| **Diabetes** |  | -0.61 | 0.542 |
| no | 26.72 (22.3, 34.1) |  |  |
| yes | 25.89 (21.5, 33.4) |  |  |
| **Atrial fibrillation** |  | -0.518 | 0.604 |
| no | 26.49 (21.7, 34.0) |  |  |
| yes | 26.16 (21.7, 33.5) |  |  |
| **Coronary artery disease** |  | -0.002 | 0.998 |
| no | 26.72 (21.65, 33.7) |  |  |
| yes | 26.13(22.2, 34.0) |  |  |
| **A1 segment development** |  | -2.889 | 0.004 |
| intact | 25.80 (21.5, 32.5) |  |  |
| Hypoplasia/absence | 29.01 (24.4, 37.8) |  |  |
| **IVT** |  | -1.358 | 0.175 |
| no | 27.3 (22.0, 35.5) |  |  |
| yes | 26.18 (21.6, 31.6) |  |  |
| **HT** |  | -7.375 | <.001 |
| no | 23.95 (20.5, 29.1) |  |  |
| yes | 33.84 (26, 40) |  |  |
| Data are expressed as median (Q1, Q3); Z: Mann–Whitney test.  FIV_BG_: final infarct volume in basal ganglia; EVT: Endovascular thrombectomy; IVT: intravenous thrombolysis; HT: hemorrhagic transformation. | | | |

| **Supplementary** **Table 2. Spearman’s correlation analysis between baseline characteristics and FIV_BG_** | | |
| --- | --- | --- |
| **Variable** | **Spearman’s rho coefficient** | ***p* value** |
| **Age** | 0.104 | 0.111 |
| **Baseline NIHSS** | 0.524 | <0.01 |
| **Collateral score** | **-0.103** | **0.115** |
| **Symptom-onset to final recanalization (min)** | 0.021 | 0.749 |
| FIV_BG_: final infarct volume in basal ganglia; NIHSS: National Institutes of Health Stroke Scale. | | |

| **Supplementary** **Table 3. Hemorrhagic transformation after reperfusion** | | | | |
| --- | --- | --- | --- | --- |
| **Characteristic** | **Hemorrhagic transformation** | | ***p* value** |  |
|  | **NO** | **YES** |  |  |
|  | **n = 144** | **n = 92** |  |  |
| **Sex, female** | 63 (43.75) | 38 (41.30) | 0.711 |  |
| **Age (years)** | 71 (62.3, 82) | 72 (62.0, 81.5) | 0.986 |  |
| **Risk factors** |  |  |  |  |
| **Hypertension** | 81 (56.25) | 61(66.30) | 0.127 |  |
| **Diabetes** | 62 (43.06) | 42 (45.65) | 0.695 |  |
| **Atrial fibrillation** | 53 (36.81) | 37 (40.22) | 0.599 |  |
| **Coronary artery disease** | 35 (24.31) | 19 (20.65) | 0.515 |  |
| **A1 hypoplasia/absence** | 12 (8.33) | 24 (26.09) | <0.01 |  |
| **Baseline NIHSS** | 14 (10,17) | 16 (14, 20) | <0.01 |  |
| **Collateral score** | **2 (1, 2)** | **2 (1, 2)** | **0.197** |  |
| **IVT** | 63 (43.75) | 28 (30.43) | 0.04 |  |
| **Symptom-onset to final recanalization (min)** | 290 (270, 315) | 290 (270, 315) | 0.871 |  |
| **Post-EVT ASPECTS_BG_** | 2(1, 2) | 1 (1, 2) | 0.017 |  |
| **FIV_BG_ (**cm^3^**)** | 23.945 (20.5, 29.1) | 33.84 (26, 40) | <0.01 |  |
| Data are expressed as n (%); Median (Q1, Q3); | | | | |
| ASPECTS_BG_: Alberta Stroke Program Early CT Score of BG regions; NIHSS: National Institutes of Health Stroke Scale; IVT: intravenous thrombolysis; EVT: Endovascular thrombectomy; FIV_BG_: final infarct volume in basal ganglia. | | | | |

| **Supplementary Table 4. Sensitivity Analysis for Predictors of HT following EVT Using Firth Penalized Logistic Regression with Bootstrap Resampling** | | |
| --- | --- | --- |
| **Variable** | ***p* value** | **OR (95% CI)** |
| **Hypoplastic/absent A1** | 0.014 | 2.944 (1.244–6.963) |
| **FIV_BG_** | <0.01 | 1.191 (1.123–1.263) |
| **Post-EVT ASPECTS_BG_** | 0.045 | 1.744 (1.013–3.001) |
| **Baseline NIHSS** | 0.656 | 0.985 (0.921–1.053) |
| **IVT** | 0.219 | 0.661 (0.342–1.279) |
| Odds ratios (OR) and 95% confidence intervals (CI) were estimated using Firth penalized logistic regression with 1,000 bootstrap replications.  FIV_BG_: final infarct volume in basal ganglia; EVT: Endovascular thrombectomy; ASPECTS_BG_: Alberta Stroke Program Early CT Score of BG regions; NIHSS: National Institutes of Health Stroke Scale; IVT: intravenous thrombolysis; HT: hemorrhagic transformation. | | |

| **Supplementary Table 5. Sensitivity Analysis of Risk Factors Associated With FIV_BG_ after excluding patients with absent A1 segment** | | | | |
| --- | --- | --- | --- | --- |
| **Variable** | **B** | **t value** | ***p* value** | **95% CI** |
| **Hypoplastic A1** | 6.425 | 4.177 | <0.01 | 3.394–9.457 |
| **Baseline NIHSS** | 0.831 | 8.904 | <0.01 | 0.647–1.015 |
| **Symptom-onset to final recanalization (min)** | 0.027 | 1.746 | 0.082 | -0.004–0.058 |
| This sensitivity analysis excluded **5** patients with absent A1 segment.  Dependent variable: FIV_BG_; R^2^ = 0.304, Adjusted R^2^ = 0.295, F = 33.066, *p* = 0.000; FIV_BG_: final infarct volume in basal ganglia; NIHSS: National Institutes of Health Stroke Scale. | | | | |

| **Supplementary Table 6. Sensitivity Analysis for Predictors of HT Following EVT after excluding patients with absent A1 segment** | | |
| --- | --- | --- |
| **Variable** | ***p* value** | **OR (95% CI)** |
| **Hypoplastic A1** | 0.021 | 2.959 (1.176–7.442) |
| **FIV_BG_** | <0.01 | 1.199 (1.129–1.275) |
| **Post-EVT ASPECTS_BG_** | 0.044 | 1.763 (1.016–3.061) |
| **Baseline NIHSS** | 0.604 | 0.982 (0.917–1.052) |
| **IVT** | 0.17 | 0.622 (0.316–1.225) |
| This sensitivity analysis excluded **5** patients with absent A1 segment.  FIV_BG_: final infarct volume in basal ganglia; EVT: Endovascular thrombectomy; ASPECTS_BG_: Alberta Stroke Program Early CT Score of BG regions; NIHSS: National Institutes of Health Stroke Scale; IVT: intravenous thrombolysis; HT: hemorrhagic transformation. | | |

| **Supplementary Table 7. Sensitivity Analysis of Risk Factors Associated with FIV_BG_ after excluding patients with follow-up NCCT** | | | | |
| --- | --- | --- | --- | --- |
| **Variable** | **B** | **t value** | ***p* value** | **95% CI** |
| **Hypoplastic A1** | 6.791 | 4.415 | <0.01 | 3.758–9.824 |
| **Baseline NIHSS** | 0.853 | 8.429 | <0.01 | 0.653–1.052 |
| **Symptom-onset to final recanalization (min)** | 0.027 | 1.587 | 0.114 | -0.007–0.061 |
| This sensitivity analysis excluded **27** patients with follow-up NCCT for FIV_BG_ assessment.  Dependent variable: FIV_BG_; R^2^ = 0.325, Adjusted R^2^ = 0.315, F = 32.874, *p* = 0.000; FIV_BG_: final infarct volume in basal ganglia; NIHSS: National Institutes of Health Stroke Scale. | | | | |

| **Supplementary Table 8. Sensitivity Analysis for Predictors of HT Following EVT after excluding patients with follow-up NCCT** | | |
| --- | --- | --- |
| **Variable** | ***p* value** | **OR (95% CI)** |
| **Hypoplastic A1** | 0.033 | 2.883 (1.090–7.622) |
| **FIV_BG_** | <0.01 | 1.258 (1.165–1.357) |
| **Post-EVT ASPECTS_BG_** | 0.013 | 2.349 (1.196–4.616) |
| **Baseline NIHSS** | 0.781 | 0.989 (0.913–1.071) |
| **IVT** | 0.13 | 0.565 (0.270–1.182) |
| This sensitivity analysis excluded **27** patients with follow-up NCCT for FIV_BG_ assessment.  FIV_BG_: final infarct volume in basal ganglia; EVT: Endovascular thrombectomy; ASPECTS_BG_: Alberta Stroke Program Early CT Score of BG regions; NIHSS: National Institutes of Health Stroke Scale; IVT: intravenous thrombolysis; HT: hemorrhagic transformation; NCCT: non-enhanced CT. | | |

| **Supplementary Table 9.** **Mediation analysis of hemorrhagic transformation** | | | |
| --- | --- | --- | --- |
| **Parameters** | **B** | **SE** | **95% CI** |
| Indirect effect | 0.868 | 0.297 | 0.340–1.507 |
| Direct effect | 1.103 | 0.439 | 0.244–1.963 |
| Total effect | 1.971 | 0.53 | 0.932–3.010 |
| **Hemorrhagic transformation as the dependent variable, A1 development as the independent variable, and FIV_BG_ as the mediator.** | | | |
